# Detection of mosaic chromosomal alterations in children with severe developmental disorders recruited to the DDD study

**DOI:** 10.1016/j.gimo.2023.100836

**Published:** 2023

**Authors:** Ruth Y. Eberhardt, Caroline F. Wright, David R. FitzPatrick, Matthew E. Hurles, Helen V. Firth

**Affiliations:** 1 Wellcome Sanger Institute, Wellcome Genome Campus, Cambridge, Hinxton, United Kingdom; 2 University of Exeter Medical School, Institute of Biomedical and Clinical Science, Royal Devon and Exeter Hospital, Exeter, United Kingdom; 3 Cambridge University Hospitals NHS Foundation Trust, Clinical Genetics, Addenbrooke's Hospital, Cambridge, United Kingdom; 4 MRC Human Genetics Unit, Institute of Genetics and Cancer, University of Edinburgh, Western General Hospital, Edinburgh, United Kingdom

**Keywords:** Anueploidy, Chromosomal alterations, Developmental disorders, Mosaic, Uniparental disomy

## Abstract

**Purpose:**

Structural mosaicism has been previously implicated in developmental disorders. We aimed to identify rare mosaic chromosomal alterations (MCAs) in probands with severe undiagnosed developmental disorders.

**Methods:**

We identified MCAs in genotyping array data from 12,530 probands in the Deciphering Developmental Disorders study using mosaic chromosome alterations caller (MoChA).

**Results:**

We found 61 MCAs in 57 probands, many of these were tissue specific. In 23 of 26 (88.5%) cases for which the MCA was detected in saliva in which blood was also available for analysis, the MCA could not be detected in blood. The MCAs included 20 polysomies, comprising either 1 arm of a chromosome or a whole chromosome, for which we were able to show the timing of the error (25% mitosis, 40% meiosis I, and 35% meiosis II). Only 2 of 57 (3.5%) of the probands in whom we found MCAs had another likely genetic diagnosis identified by exome sequencing, despite an overall diagnostic yield of ∼40% across the cohort.

**Conclusion:**

Our results show that identification of MCAs provides candidate diagnoses for previously undiagnosed patients with developmental disorders, potentially explaining ∼0.45% of cases in the Deciphering Developmental Disorders study. Nearly 90% of these MCAs would have remained undetected by analyzing DNA from blood and no other tissue.

## Introduction

Genetic mosaicism is the presence of 2 or more genetically distinct lineages of cells in 1 individual, arising from post-zygotic variants. Mosaic variation can consist of single-nucleotide variants (SNVs) and indels, or it may involve larger stretches of the genome, including copy number variants (CNVs) and aneuploidies. Mosaicism has been associated with diseases including neurodevelopmental disorders.^1^, ^2^, ^3^, ^4^ The clinical consequences of mosaicism vary according to the nature of the mosaic event, the stage of development at which this event occurs, and the tissue types in which this event is present.^5^

Very large chromosomal abnormalities, such as complete autosomal aneuploidy, are generally incompatible with life, with the exception of trisomy 21. However, mosaic aneuploidies are better tolerated and have been identified in many autosomes, including chromosomes 7, 8, 9, 14, 16, 17, 19, and 22.^6^, ^7^, ^8^ Moreover, children with mosaic trisomies of chromosomes 13 and 18 live for much longer than those with constitutive trisomies, with 80% and 70% of patients with mosaic trisomy 13 and 18, respectively, surviving for at least a year compared with 8% with non-mosaic trisomies.^9^^,^^10^ Additionally, mosaic uniparental disomy (UPD, in which 2 copies of 1 chromosome are inherited from 1 parent) has also been associated with several developmental disorders.^5^^,^^11^, ^12^, ^13^, ^14^

Mosaic chromosomal alterations (MCAs) can be detected in SNP genotyping array data by identifying differences from the expected log R ratio (LRR) and B-allele frequency (BAF). LRR gives a measure of the intensity at any given position on the array and deviations from the expected LRR indicate an abnormal copy number. BAF is a normalized measure of the intensity ratio of 2 alleles (A and B), such that a BAF of 1 or 0 indicates the complete absence of 1 of the 2 alleles (eg, homozygous AA or BB), and a BAF of 0.5 indicates the equal presence of both alleles (eg, heterozygous AB). Deviations in BAF can indicate the presence of CNVs or UPD.

There are several tools that use LRR and BAF to detect MCAs from genotyping array data. Mosaic alteration detection (MAD) uses the genome alteration detection algorithm to detect mosaic CNVs and UPDs.^15^^,^^16^ Parent-of-origin-based detection in trios (triPOD) uses an overlapping window approach to detect mosaic CNVs and UPDs in parent-offspring trios, but the absence of parental data makes this tool unsuitable for many cohorts.^17^ MONTAGE is a recently developed tool using a sliding window approach for rapid detection of mosaic CNVs; however, it is unable to detect mosaic UPDs.^18^ MoChA is a bcftools plugin, which identifies mosaic CNVs and UPDs in array data using a hidden Markov model to detect imbalances in phased BAF and LRR.^19^^,^^20^ We chose to use MoChA because it is quick to run, sensitive, does not require trio information, and is able to detect both mosaic CNVs and UPDs.

The Deciphering Developmental Disorders study (DDD) is a cohort of 13,612 children with severe developmental disorders.^21^ The DDD study recruited patients from 2011 to 2015 who remained undiagnosed after expert review by a clinical geneticist and completion of routine genetic testing. These patients had neurodevelopmental disorders, congenital anomalies, abnormal growth parameters, dysmorphic features, and genetic disorders of significant impact for which the molecular basis was unknown. 58.4% of these patients were male and the median age at recruitment was 7 years (range 0-63 years). Approximately 85% had undergone array analysis (CMA) before recruitment, often supplemented by phenotype-targeted gene sequencing, but remained without a molecular genetic diagnosis to explain their phenotypes. MCAs have previously been investigated in this cohort using exome sequencing (ES) from 4,911 probands using MrMosaic and additionally from SNP genotyping array data for 1,303 of these probands using MAD and triPOD.^3^^,^^4^ However, the majority of DDD probands have not been analyzed systematically for the presence of MCAs. Here, we used MoChA to detect MCAs across all 12,530 probands with SNP genotyping array data in the DDD study.

## Materials and Methods

### Patient cohort

A total of 13,612 probands with developmental disorders, and their parents, were recruited to the DDD study. Blood-extracted DNA and/or saliva samples were collected from all probands, and, where possible, saliva samples were collected from parents. SNP genotyping array data were generated for 12,530 of the probands in this study. Probands were systematically phenotyped by consultant clinical geneticists using the Human Phenotype Ontology^22^ and a structured questionnaire in DECIPHER (www.deciphergenomics.org).^23^

### MCA detection from array

Samples from 1465 probands were genotyped on the Illumina HumanOmniExpress chip, and samples from 11,065 probands were genotyped on the Illumina HumanCoreExome chip. Intensity data were converted into VCF (variant call format), including BAF and LRR, using gtc2vcf.^24^

MCAs were detected using MoChA.^19^^,^^20^ The output was filtered to remove samples with the following: BAF phase concordance across phased heterozygous sites underlying the call of >0.51, calls <100 kbp, calls with a LOD score of <10 for the model based on BAF and genotype phase, calls flagged by MoChA as likely germline CNVs, and calls with an estimated cell fraction of >50%. More stringent filters were subsequently applied to identify rare MCAs of likely clinical significance: events that occur in more than 1% of the cohort were removed, events overlapping CNVs previously identified in the cohort were removed, and events that were <1 Mb in length were removed unless they overlapped genes known to cause developmental disorders (https://www.ebi.ac.uk/gene2phenotype).^25^ All MCAs remaining after these filters were manually reviewed to evaluate data quality; events with low deviation in BAF, events in regions where the genotyping array had sparse SNPs and events in samples with noisy data were removed.

## Results

### Potentially clinically relevant MCAs were identified in 57 probands with developmental disorders

**Figure 1:**
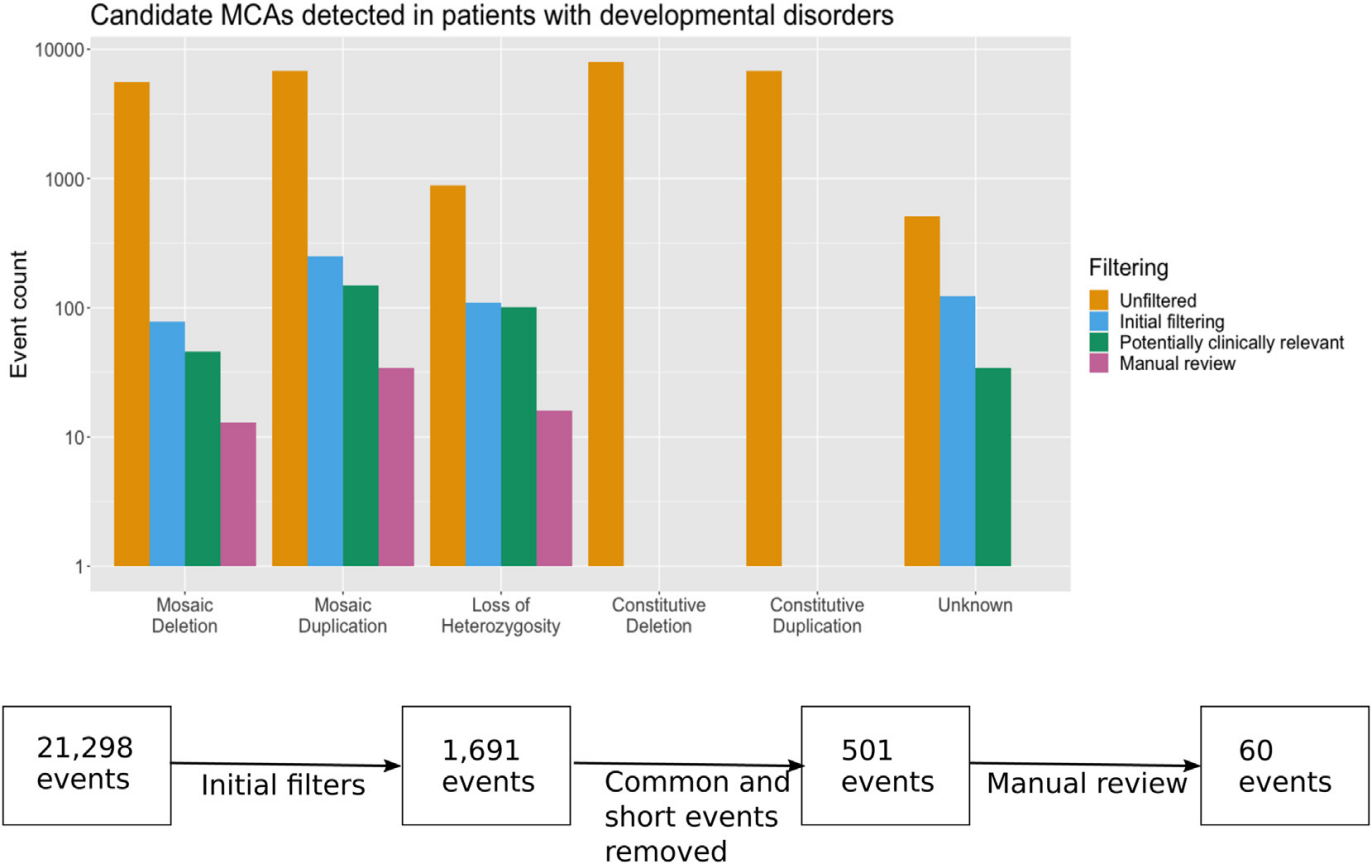
**Workflow used to identify potentially clinically relevant MCAs.** The flowchart shows the different filtering stages and the total number of events remaining at each stage. The bar plot shows the number of events of each type remaining at each stage.

**Figure 2:**
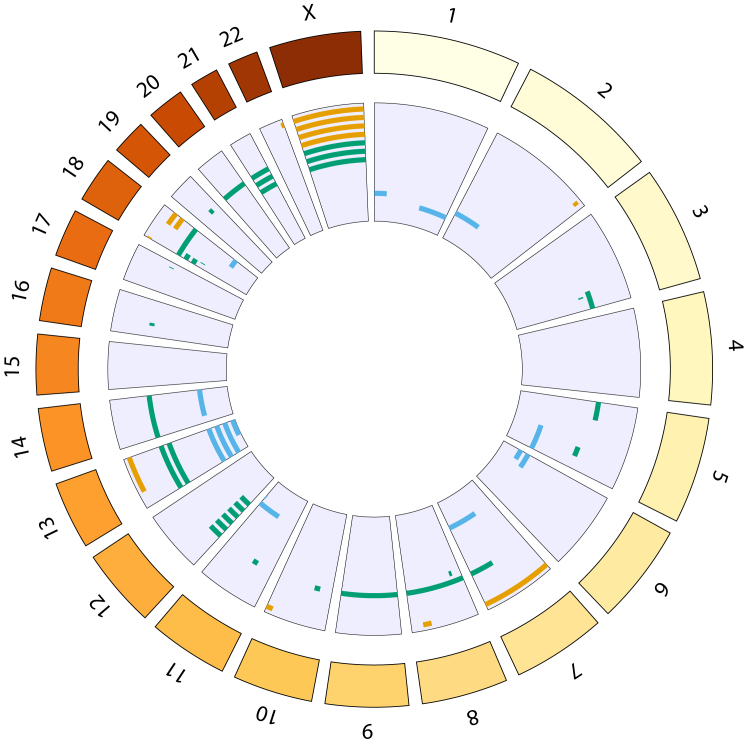
**The distribution of MCAs in the genome.** Each bar represents 1 event; deletions are shown in orange, duplications in green, and loss of heterozygosity in blue.

A total of 28,864 candidate MCAs were identified by MoChA in the 12,530 probands studied. It was our intention to identify potentially clinically relevant MCAs and our stringent filtering will inevitably have discarded some true positive MCAs. Besides the filtering recommended by the authors of the MoChA software, we filtered on size, frequency, and visibility to the naked eye of deviation on plots of BAF and LRR. Short MCAs, common MCAs, and those invisible to the naked eye because of low deviation in BAF are less likely to be pathogenic. After initial filtering (Methods), 558 candidate MCAs remained. These events comprised 249 duplications, 78 deletions, 109 copy number neutral loss-of-heterozygosity events, and 122 events in which the type could not be determined. After further filtering to identify events of potential clinical relevance, 330 events were reviewed manually to evaluate data quality, and, subsequently, 61 events from 57 probands were identified for clinical evaluation (Figure 1, Supplemental Table 1). These MCAs represent a potential diagnostic yield of 0.45% in our cohort. These 61 events comprise 33 duplications, 12 deletions, 14 copy number neutral loss-of-heterozygosity events, a deletion flanked by copy number neutral loss of heterozygosity, and a duplication followed by UPD of the majority of the q-arm of chromosome 1. The 33 duplications affect 18 different chromosomes, with the most frequently affected being chromosome 12 (6 events), the 12 deletions affect 7 different chromosomes, of which the most frequently affected is chromosome X (4 events), and the UPDs affect 9 different chromosomes, of which the most frequently affected is chromosome 13 (4 events) (Figure 2). All of these 57 probands had previously undergone ES, but pathogenic or likely pathogenic variants had only been identified in 2 of these individuals,^26^ indicative that these MCAs are likely diagnostic findings for the developmental disorders in these individuals because no alternative diagnoses were evident.

### Tissue specificity was observed for the majority of clinically relevant MCAs

A total of 42 MCAs were detected in saliva from 38 probands (Supplemental Table 1). For 9 of the probands in which a total of 11 MCAs were found in saliva, we also had genotyping array data from blood; the MCA was also detected in only 3 of these. In an additional 14 probands that 15 MCAs were detected in saliva, we also had ES and/or array comparative genomic hybridization (aCGH) data from blood; there was no evidence of the MCA in any of these. For the remaining 16 MCAs from 15 probands, no other tissue type was available for testing. A total of 22 MCAs were detected in blood from 22 probands. There were genotyping array data available from saliva in 3 of these, and, in all 3 cases, the MCA was also detected in saliva at a cell fraction higher than in blood, eg, mosaic deletion in ID 259003 is present in saliva at 53% and blood at 32% (see Table 1).

**Table 1:** Pathogenic and likely pathogenic MCAs in DDD patients

Chr.	Event type	Start-end (GRCh37)	Size (Mb)	Blood Saliva % %	DECIPHER	Syndrome	Key Phenotypes Shown by Patients with Recurrent MCAs
1	dup+upd	165589535-249250621 (q)	83.66	nd 39	296586	Mosaic likely pathogenic duplication - ACMG score 0.90 (G1A, G3C)	

2	del	223873590-232804522	8.93	0 22	265112	Mosaic pathogenic deletion - ACMG score 1.90 (L1A, L2A, L3C)	

3	dup	153567441-198022430	44.46	0 54	258956	Mosaic pathogenic partial trisomy 3q23-ter	

5	dup	1-46174864	46.18	nd 36	305868	Mosaic trisomy 5p	
5	dup	123851734-148651711	24.80	0 34	261240	Mosaic likely pathogenic duplication - ACMG score 0.90 (G1A, G3C)	

7	del	1-159138663 (w)	159.14	58 nd	285424	MIRAGE syndrome due to *SAMD9* variant	
7	upd	64864800-159138663 (q)	94.27	7 0	275728	MIRAGE syndrome due to *SAMD9* variant	
7	dup	99227172-159138663	59.91	0 43	283385	Mosaic pathogenic partial trisomy 7q21.11-ter	

8	dup	1-146364022 (w)	146.36	45 nd	275705	Mosaic trisomy 8	
8	dup	22487087-29344462	6.85	25 nd	263580	Mosaic pathogenic duplication - ACMG score 1.35 (G1A, G2H, G3C, G5A)	
8	del	101011612-120215645	19.20	nd 33	290927	Langer Gideon syndrome (includes EXT1)	

9	dup	1-141213431 (w)	141.21	19 nd	295318	Mosaic trisomy 9	

10	del	121375181-135534747	14.16	0 43	274013	Mosaic pathogenic deletion - ACMG score 1.35 (L1A, L2C, L5A)	

11	dup	41887927-47877800	5.99	0 22	259029	Mosaic pathogenic duplication - ACMG score 1.35 (G1A G3C, G5A)	

12	dup	1-26749137 (p)	26.75	0 33	280908	Pallister-Killian syndrome	) )
12	dup	1-34523378 (p)	34.52	0 34	283911	Pallister-Killian syndrome	)
12	dup	1-34758266 (p)	34.76	0 63	299715	Pallister-Killian syndrome	GDD, irregular pigmentation, sparse scalp hair
12	dup	1-34781187 (p)	34.78	0 44	261373	Pallister-Killian syndrome	)
12	dup	1-34801271 (p)	34.80	0 25	286521	Pallister-Killian syndrome	)
12	dup	1-34826574 (p)	34.83	0 63	265800	Pallister-Killian syndrome	)

13	dup	1-115169878 (w)	115.17	0 11	264072	Mosaic trisomy 13	) Congenital heart disease and GDD
13	dup	1-115169878 (w)	115.17	16 nd	283167	Mosaic trisomy 13	)
13	del flanked by loh	33986219-115169878	81.18	23 nd	293046	Mosaic pathogenic deletion - ACMG score 2.35 (L1A, L2A, L3C, L5A)	

14	dup	1-107349540 (w)	107.35	nd 45	306061	Mosaic trisomy 14	
14	upd (pat)	1-107349540 (w)	85.62	nd 36	303525	Mosaic Kagami-Ogata syndrome	

16	dup	27183151-34747045	7.56	0 31	263654	Mosaic pathogenic duplication - ACMG score 1.35 (G1A, G2H, G3C, G5A)	

18	dup	1-14084928 (p)	14.09	nd 16	266471	Mosaic pathogenic duplication - ACMG score 1.35 (G1A, G2H, G3C, G5A)	) GDD ,hypotonia
18	dup	1-14988113	14.99	0 25	273553	Mosaic pathogenic duplication - ACMG score 1.35 (G1A, G2H, G3C, G5A)	)
18	dup	1-78077248 (p)	78.08	14 nd	260037	Mosaic partial trisomy 18	
18	del	48368256-78077248	29.71	0 45	260462	Mosaic partial monosomy 18q12.3-ter	) Microcephaly, hypotonia
18	del	49315539-78077248	28.76	0 49	274600	Mosaic partial monosomy 18q12.3-ter	)

19	dup	31740744-41525952	9.79	nd 29	290927	Mosaic pathogenic duplication - ACMG score 1.35 (G1A, G2H, G3C, G5A)	

20	dup	1-63025520 (w)	63.03	0 22	258190	Mosaic trisomy 20	

21	dup	1-48129895 (w)	48.13	nd 12	294112	Mosaic trisomy 21	)
21	dup	1-48129895 (w)	48.13	nd 40	301048	Mosaic trisomy 21	GDD, delayed speech and language, sandal gap
21	dup	1-48129895 (w)	48.13	nd 30	306282	Mosaic trisomy 21	)

22	del	45311891-51304566	5.99	32 53	259003	Mosaic pathogenic deletion - ACMG score 1.45 (L1A, L2A, L3A, L5A)	

X	dup	1-155270560 (w)	155.27	3 nd	287504	Mosaic XXXY/XXXXY syndrome	
X	dup	38541235-41749282	3.21	19 nd	280407	Mosaic triple X syndrome	) Cognitive impairment
X	dup	38557085-41742515	3.19	15 nd	277716	Mosaic triple X syndrome	)

X	del	1-155270560 (w)	155.27	0 43	283385	Mosaic Turner syndrome	)
X	del	1-155270560 (w)	155.27	nd 55(p) 24(q)	291029	Mosaic Turner syndrome	) Short stature
X	del	1-155270560 (w)	155.27	25 nd	291198	Mosaic Turner syndrome	)
X	del	1-155270560 (w)	155.27	nd 28	300814	Mosaic Turner syndrome	)

*Chr.,* chromosome; *del*, deletion; *dup*, duplication; *GDD*, global developmental delay; *MCA*, mosaic chromosomal alterations; *nd*, not done; *p*, p-arm; *q*, q-arm; *upd*, uniparental disomy; *upd (pat)*, paternal uniparental disomy; *w*, whole chromosome.

### Mosaic aneuploidy can originate in mitosis, meiosis I or meiosis II

**Figure 3:**
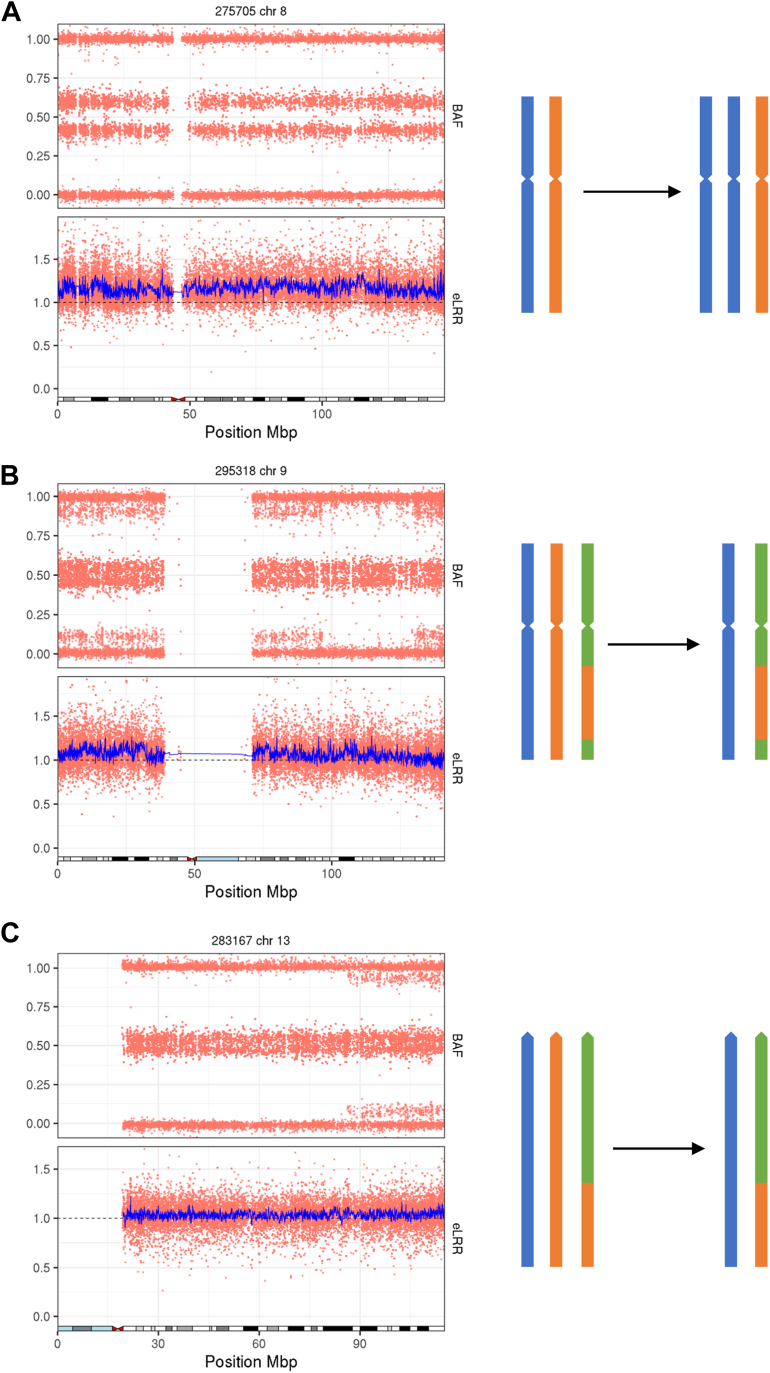
**Origin of mosaic trisomies.** A. A mosaic trisomy that has occurred during mitosis. B. A mosaic trisomy that has occurred during meiosis I. C. A mosaic trisomy that has occurred during meiosis II.

The 33 observed duplications include 20 polysomies, 11 of which affect a whole chromosome and 9 of which consist of the p-arm only (Supplemental Table 1). Ten different chromosomes were affected by these polysomies (5, 8, 9, 12, 13, 14, 18, 21, 20, and X). The origin of a trisomy can be determined by examination of the BAF pattern.^6^ The absence of a third haplotype indicates that 5 of these events (3 in chromosome 12 p-arm, 1 in chromosome 8, and 1 in chromosome X) have a mitotic origin. Eight events (1 in chromosome 5 p-arm, 1 in each of chromosomes 9, 13, 14, 18, and 20, and 2 in chromosome 21) have a BAF pattern consistent with occurrence in meiosis I, where 3 haplotypes are observed near to the centromere. A pattern consistent with occurrence in meiosis II, with additional haplotypes present at the telomeres but not at the centromere, is observed in the remaining 7 cases (3 chromosome 12 p-arm, 2 chromosome 18 p-arm, 1 chromosome 13, and 1 chromosome 21) (Figure 3).

The 12 observed deletions include 4 mosaic monosomies, 1 in chromosome 7, and 3 in the X chromosome. One of the X chromosome monosomies has mosaic monosomy of the p-arm in 50% of cells and mosaic monosomy of the q-arm in 25 % of cells (Supplemental Figure 1). In all of the observed monosomies, the BAF patterns are consistent with origination via mitotic nondisjunction, with 2 distinct haplotypes observed, rather than monosomy rescue.^6^

The 14 mosaic copy number neutral loss-of-heterozygosity events identified include 5 mosaic UPDs that comprise all or most of 1 arm of a chromosome (1q, 2p, 6p, 7q, and 11q), 4 mosaic UPDs that affect an entire chromosome (3 in chromosome 13 and 1 in chromosome 14), and 5 smaller loss-of-heterozygosity events. In 1 case, the UPD in the p-arm of chromosome 6 shows 2 different clonalities and is therefore likely to have arisen as 2 different events (Supplemental Figure 2). Furthermore, we detected complex chromosomal events in several probands, including the following: deletion flanked by copy number neutral loss-of-heterozygosity, spanning a total of 80.9 Mb in chromosome 13 (Supplemental Figure 3A), a duplication followed by UPD of the majority of chromosome 1 q-arm (Supplemental Figure 3B), and a patient with a 59.9 Mb duplication in chromosome 7, mosaic polysomy of the first 95 Mb of chromosome X and non-mosaic polysomy of the remainder of chromosome X (Supplemental Figure 3C). We were unable to determine the origin of these events.

## Discussion

Using these filters, we have identified MCAs of interest using genotyping array data in 57 of 12,530 (0.45%) probands with severe developmental disorders. Fifty-four patients had single events, 2 had 2 independent events, and 1 had 3 MCA events. Our findings are consistent with Sherman et al in which 46 mosaic CNVs were identified in 12,077 probands with autism spectrum disorder (ASD).^27^ After ES, only 2 (3.5%) patients with potentially clinically significant MCAs identified here have previously identified pathogenic or likely pathogenic SNVs, indels, or CNVs. Compared with the cohort-wide diagnostic yield in the DDD study of ∼40%,^28^ the observed enrichment of undiagnosed patients in this group suggests that most of these MCAs are diagnostic.

Clinical evaluation of the phenotypic and genomic data by an experienced clinical geneticist resulted in 44 diagnoses of MCAs that were either well-established pathogenic variants, eg, Mosaic tetrasomy 12p in Pallister-Killian syndrome, or where the chromosomal anomaly was classified as Pathogenic or Likely Pathogenic using the ACMG^TM^ CNV classifier^29^ (Table 1). These comprised mosaic polysomies of chromosomes 12p, 18p, and 20, mosaic duplications of chromosomes 5, 8, 11, and 17, mosaic deletions of chromosomes 2 and 22, mosaic loss of heterozygosity in chromosome 5, and a mosaic deletion-duplication-deletion in chromosome 18. Clinical features indicative of a mosaic event, including abnormalities of skin pigmentation, syndactyly, and/or asymmetry, were observed in only 7 of the 44 probands with a diagnostic finding. The remainder of the MCAs were interpreted to be variants of uncertain significance. Recruitment to DDD was by ∼200 experienced consultant clinical geneticists. The fact that apparently recognizable disorders, such as Pallister-Killian syndrome or mosaic trisomy 13 or 21, have been identified in this study demonstrates that clinical assessment of mosaic disorders is not entirely reliable, and it is easy for them to be overlooked in clinic.

MoChA is unable to distinguish between mosaic trisomies, mosaic tetrasomies, or other mosaic polysomies. The 6 mosaic polysomies involving chromosome 12p are likely to be Pallister-Killian syndrome, in which an isochromosome comprising 2 copies of chromosome 12p is present.^30^ We also identify a case that is likely to be mosaic tetrasomy 5p. Only 5 cases of mosaic tetrasomy 5p, in which an isochromosome consisting of 2 copies of the p-arm of chromosome is present, have been reported to date.^31^ In addition a case of an isochromosome consisting of 2 partial copies of 5p has been reported.^32^ A small number of live-born cases of mosaic isochromosome 18p have been reported in the literature;^33^, ^34^, ^35^ we identify a likely mosaic tetrasomy 18p.

Mosaic trisomy can occur by meiotic non-disjunction in the oocyte or sperm followed by trisomy rescue or by mitotic nondisjunction at a later stage of development. Using genotyping array data, we were able to distinguish between mosaic polysomies occurring via nondisjunction at mitosis or meiosis, and 15 of 20 trisomies detected (75%) were meiotic in origin. The timing of the event has implications for counseling families because some women have a higher rate of meiotic nondisjunction and therefore a greater recurrence risk.^36^^,^^37^ This estimate is somewhat higher than Conlin et al, who found that 10 of 20 (50%) of trisomies had a meiotic origin^6^ and may reflect ascertainment differences between the cohorts.

The mosaic monosomies we detected all arose by mitotic nondisjunction, rather than monosomy rescue, which would result in homozygosity. This finding has important implications for recurrence risk because for the former this is negligible, whereas the latter raises the potential for gonadal mosaicism. We were unable to detect monosomy rescue because the method used is phase-based and therefore cannot detect events in runs of homozygosity;^20^ however, Conlin et al also only reported mitotic events.^6^ Similarly, our study can only detect mosaic UPD arising from trisomy rescue and resulting in heterodisomy because any events arising from monosomy rescue will result in isodisomy and lack heterozygous regions.

Two of the MCAs described here, a mosaic monosomy and a mosaic UPD, are in chromosome 7 and include the *SAMD9* gene. In both patients pathogenic *SAMD9* variants have previously been reported. Loss of chromosome 7 and UPD of 7q have previously been described in patients with MIRAGE syndrome (MIM #617053), this is believed to be an adaptation to the growth-suppressing effect of the *SAMD9* variants.^38^^,^^39^

We found MoChA to be a highly effective tool for detecting clinically relevant MCAs. Smaller subsets of the DDD cohort have previously been analyzed for MCAs using alternative methods. Previously published analysis of structural mosaicism in genotyping arrays from 1303 DDD probands using MAD and triPOD described MCAs in 12 probands.^3^ However, neither MAD or triPOD detected all 12 of these events, and it was shown that a combination of algorithms was necessary to maximize diagnostic yield. We tested 11 of these probands and found 9 of the previously reported events. MoChA identifies events found by MAD and missed by triPOD and vice versa. One of the events missing in our filtered MoChA data set was a genome-wide paternal UPD. This event was found by MoChA; however, the sample was removed by the MoChA default filters designed to exclude samples that are either contaminated or low quality DNA based on high-phased BAF auto-correlation. The second event that is not found by MoChA was a UPD of chromosome 14 present in around two-thirds of cells; it is not clear why this event was not found by MoChA; however, no mosaic UPDs with a cell fraction of >0.4 were detected. Additionally, a duplication in chromosome 17 not previously identified using MAD and triPOD was detected using MoChA. Furthermore, the previously published analysis of structural mosaicism in ES data from 4911 DDD probands using MrMosaic described MCAs in 9 probands, all of which were detected using MoChA.^4^ In the same 4911 probands, an additional 5 events were detected using MoChA that were not detected using MrMosaic, including the following: 2 mosaic polysomies of chromosome 18p, 1 mosaic polysomy of chromosome 8, 1 mosaic UPD of chromosome 13, and 1 mosaic UPD of chromosome 2p. These results show that using more than 1 tool will increase the number of MCAs detected; however, if only a single tool is to be used (for example from a cost-benefit perspective), then MoChA is a good choice because of its high sensitivity, rapid run time, and ability to detect both mosaic CNVs and UPDs.

Importantly, 23 of 26 (88.5%) of MCAs detected from saliva in which blood was also available for testing could not be detected in blood-derived DNA. This result contrasts with our previous observation that mosaic de novo SNVs were observed at similar variant allele fractions in both blood and saliva^40^ and may suggest stronger negative selection against MCAs within blood lineages. One limitation of our study is that we only have data from 2 tissues, blood, and saliva. Although study of saliva yields more mosaic events than blood, variants occurring later in embryonic development are likely to be present in a narrower range of tissues and we may therefore miss potentially diagnostic events by not having more tissue types available to study. Nonetheless, our observations highlight the importance of testing saliva (or other tissues) where possible to avoid missing mosaic structural events.

There is currently a paucity of large-scale studies of MCAs in disease cohorts. Our results are comparable in both size and yield to those of Sherman et al, who report 46 mosaic CNVs in a cohort of 12,077 patients with autism spectrum disorder (0.38%),^27^ our MCAs included mosaic CNVs in 43 of our 12,530 patients (0.34%). Study of mosaic aneuploidies and UPDs by Conlin et al in a cohort of 2019 patients referred to the Children's Hospital of Philadelphia Clinical CytoGenomics laboratory had a higher yield than our study (30/2019, 1.5%).^6^ Our results add to this body of literature but are likely to be an underestimate of the true diagnostic yield from MCAs in developmental disorders because of under-ascertainment in the DDD study of cases who would have been previously diagnosed using prior clinical genetic testing (such as karyotyping and microarray analysis).^41^

Our results show that rare MCAs are an important source of diagnoses in severe developmental disorders. The meiotic or mitotic origin of the variant can often be determined through careful analysis of genotyping array data and has important implications for recurrence risk. This work suggests that routinely analyzing SNP genotyping array data could provide potential diagnoses that are currently difficult to detect via ES and that diagnostic yield will be increased by the analysis of saliva samples. We recommend that clinical teams consider the use of saliva-derived DNA for genotyping array analysis for the investigation of neurodevelopmental disorders to complement genome-wide sequencing using blood-derived DNA.

## Data Availability

Diagnostic variants and phenotypes for probands included in this study are available via the DECIPHER database (https://deciphergenomics.org/). Genotype array data are available in EGA.

## Conflict of Interest

M.E.H. is a co-founder and non-executive director of Congenica Ltd and an advisor to AstraZeneca. All other authors declare no conflicts of interest.
