# Supplementary figures and images for: Detection of mosaic chromosomal alterations in children with severe developmental disorders recruited to the DDD study
Source: Genet Med Open. 2023 Oct 12;1(1):100836. doi: 10.1016/j.gimo.2023.100836 (PMC11436381; doi:10.1016/j.gimo.2023.100836)

291029 chr X

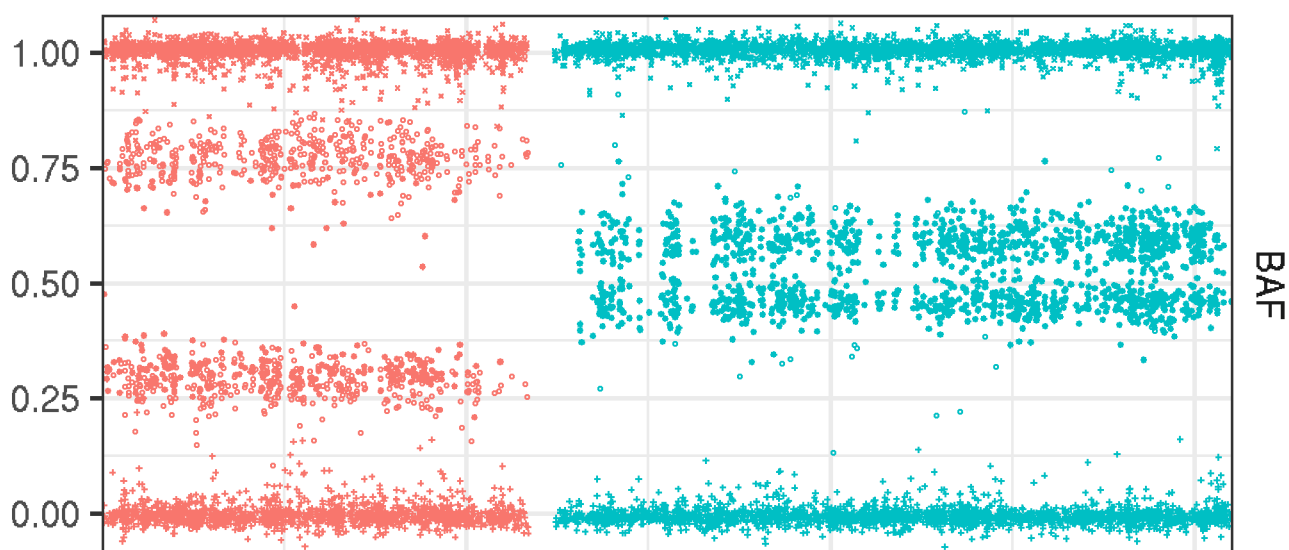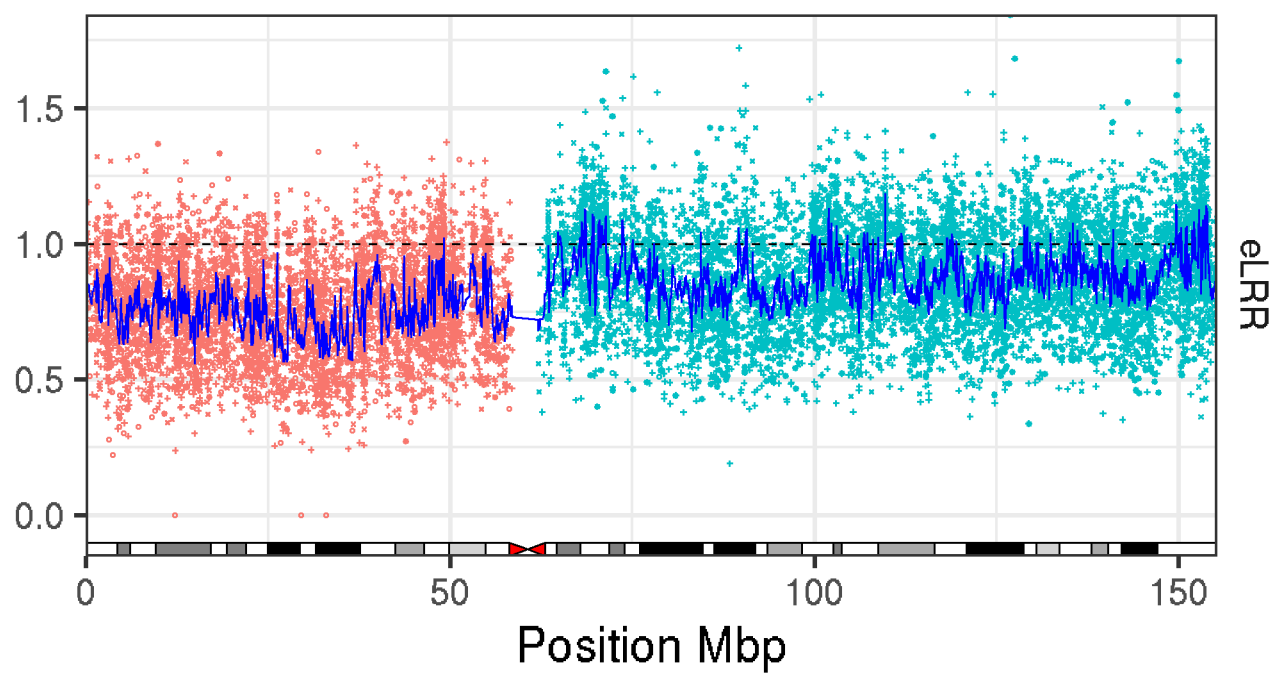

Supplement: Supplementary Figure 1 [file mmc1.pdf]

287432 chr 6

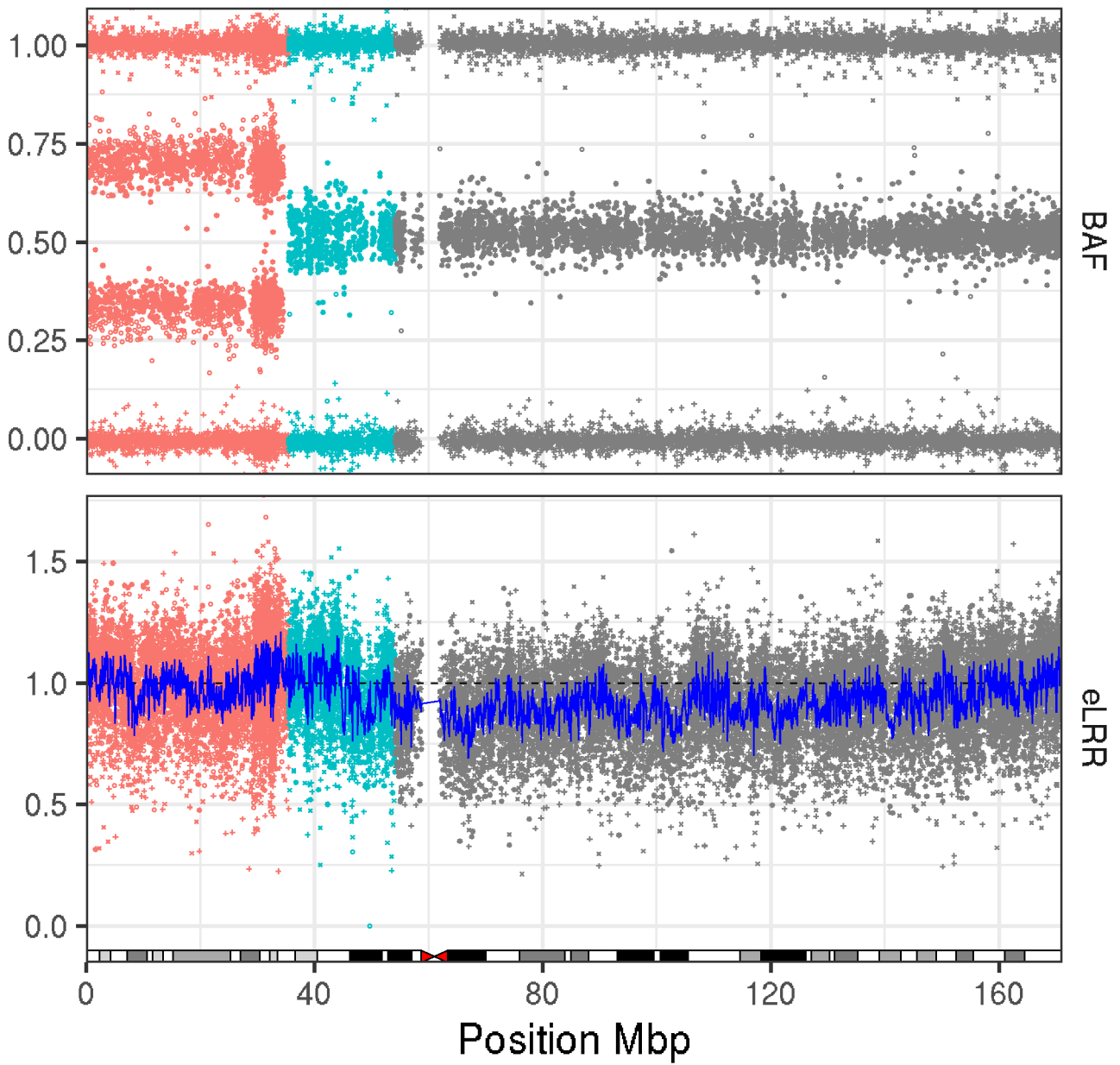

Supplement: Supplementary Figure 2 [file mmc2.pdf]

## Slide 1
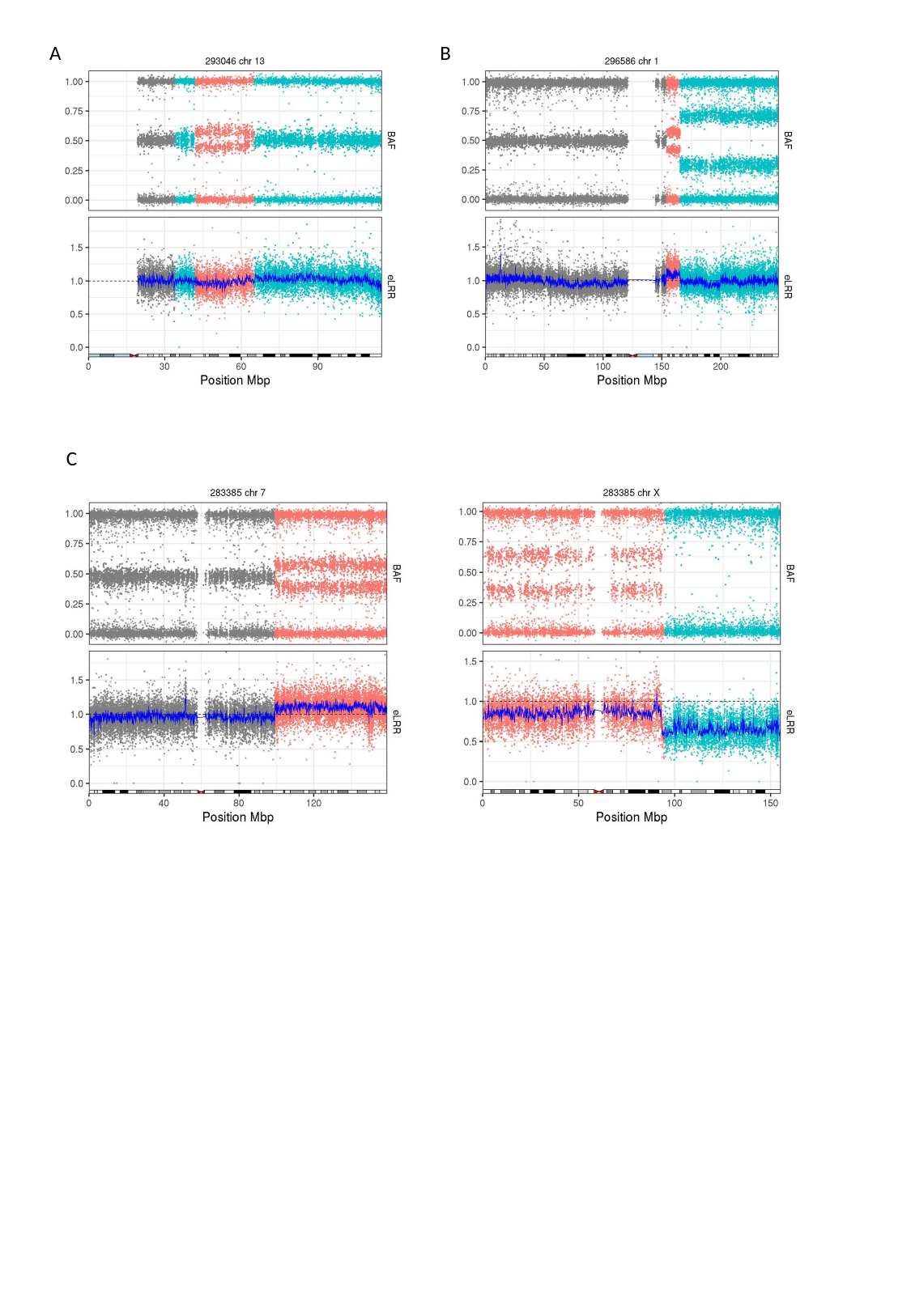

A
B
C

Supplement: Supplementary Figure 3 [file mmc3.pptx]
